# Supplementary material for: Identification of Azoxystrobin Glutathione Conjugate Metabolites in Maize Roots by LC-MS
Source: Molecules. 2019 Jul 5;24(13):2473. doi: 10.3390/molecules24132473 (PMC6651014; doi:10.3390/molecules24132473)
Supplement: Supplementary file 1 [file molecules-24-02473-s001.pdf]

Supplementary Figures

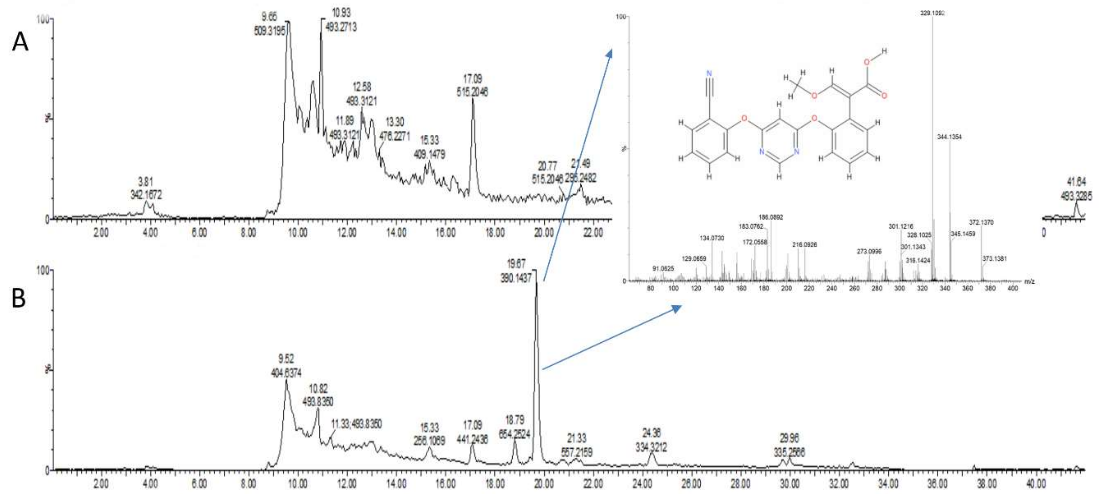

Supplementary Figure 1. Extracted MS chromatograms at MS1 level of the precursor specie 390.1 m/z eluted after 19.68 minutes and MS/MS DDA isolation and fragmentation of the same (zoom-in). The control sample untargeted MS survey profile (panel A) has been correlated (extracted chromatogram function performed by MassLynx Software) to the azoxystrobin treated ones (panel B) for the peaks containing the precursor specie 390.1 m/z. At 19.68 minutes as elution time, the only differential peak at 390.1 m/z containing the MS/MS profile with degradation products of azoxystrobin (demethylated) was detected in the treated samples roots (panel B).

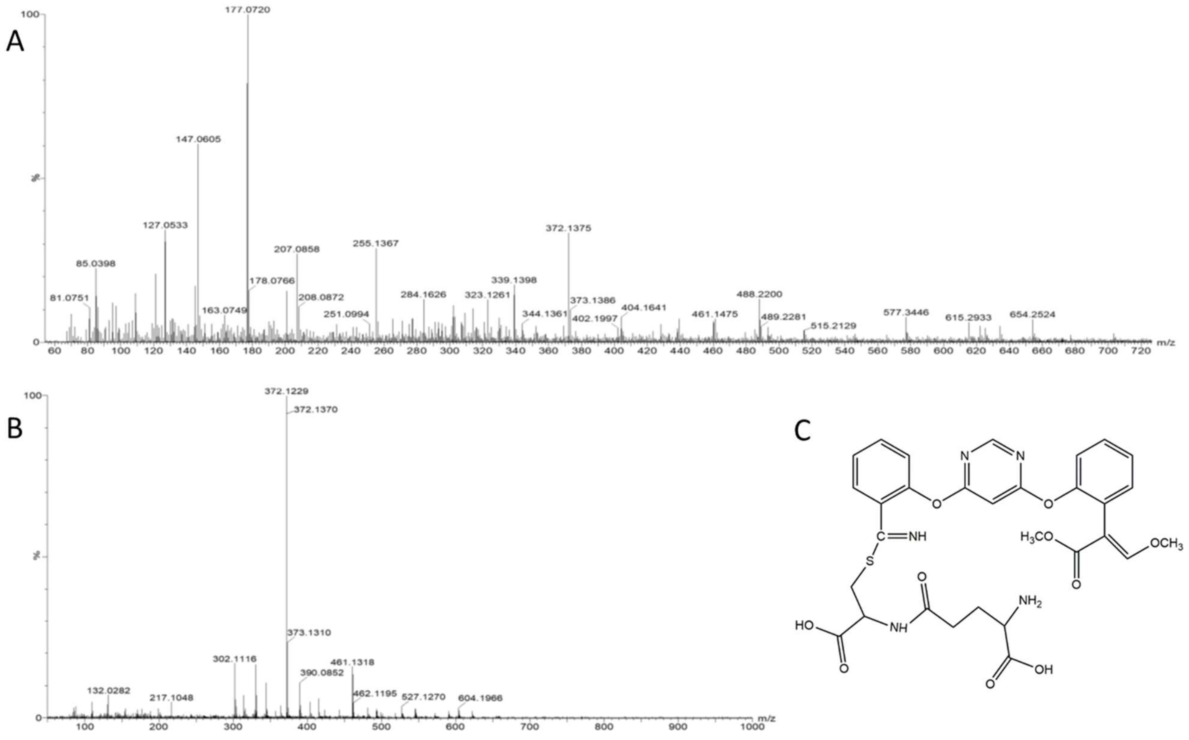

Supplementary Figure 2. Comparison of MSe and MS/MS DDA spectra of the precursor specie 654 m/z. Possible identification of GSH azoxystrobin conjugate at RT 18.67 min. The analysis of the MSe (all ions fragmentation, panel A) and MS/MS profile (DDA, panel B) agree that the precursor of this azoxystrobin-GSH conjugate could be the compound with 654 m/z (panel C) represented glycil neutral loss (57) of the full GSH-azoxystrobin conjugate (see Figure 6). Besides, the azoxystrobin signature is confirmed by the presence of 372 m/z and other specific fragments in MSe mode and in the spectra of isolated MS/MS DDA specie with 654.19 m/z.

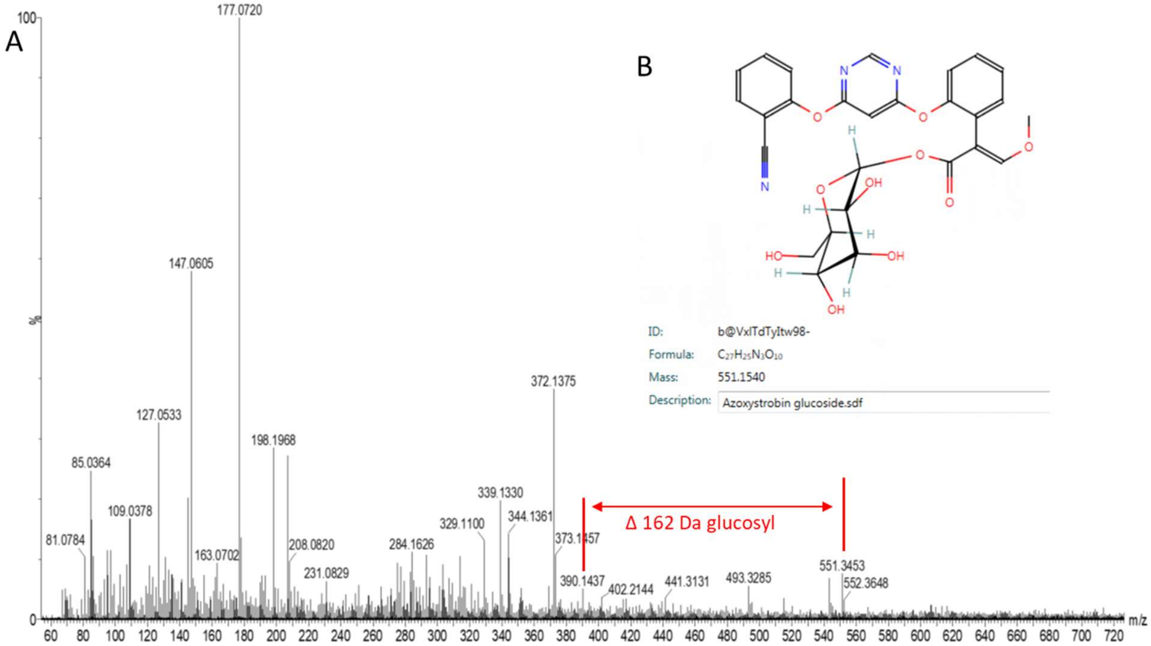

Supplementary Figure 3. Interpretation of MSe spectra of the precursor specie 552 m/z. Possible identification of potential azoxystrobin conjugate at RT 19.72 min. The analysis of this all ion fragmentation profile reveals that the precursor of this azoxystrobin-conjugate/metabolite could be the compound with mass 551.15 (glycosylated axozystrobin, panel B) as confirmed by the presence of 372.13 m/z and the presence of 390.14 m/z as glucosyl neutral loss (delta 162). Such evidence could account for the fact that this precursor could be azoxystrobin free acid glucoside, also validated by DDA MS/MS fragment analysis of the isolated 552 m/z specie (data not shown).

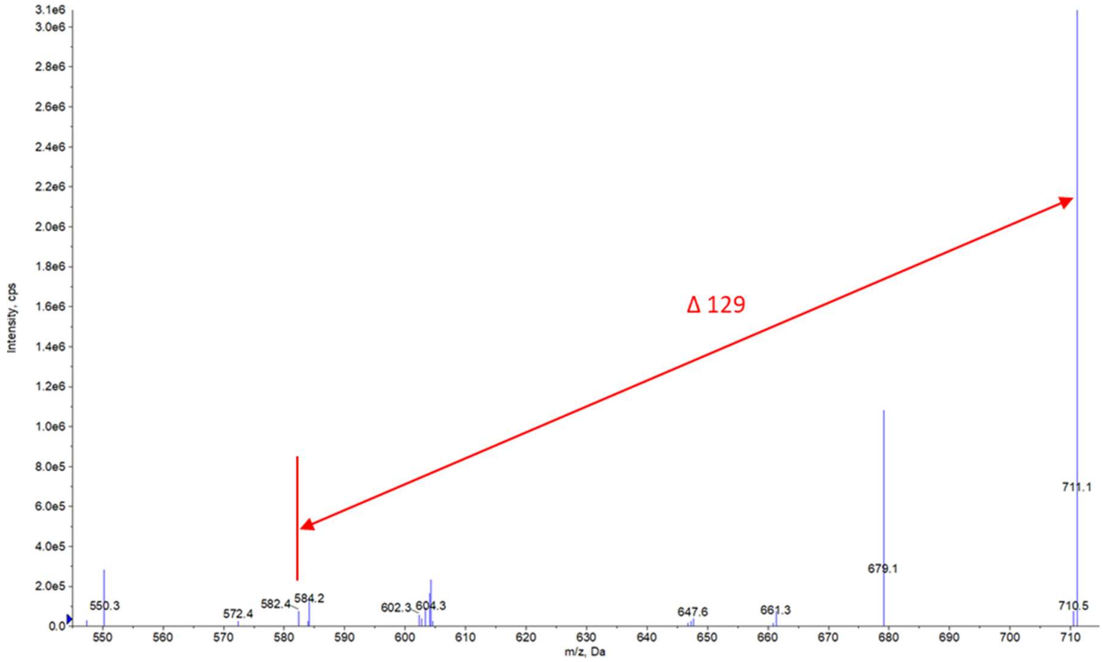

Supplementary Figure 4. QTRAP generated MS/MS spectra of 771 m/z peak eluting at RT 18.10-18.36 minutes from nano-LC-Q-TOF. Using LC-C18 coupled to QQQ it was possible to detect from the isolated 711 m/z specie a neutral loss of 129 (product ion 582 m/z) corresponding to the loss of glutamic acid residue from glutathione-azoxystrobin conjugate.

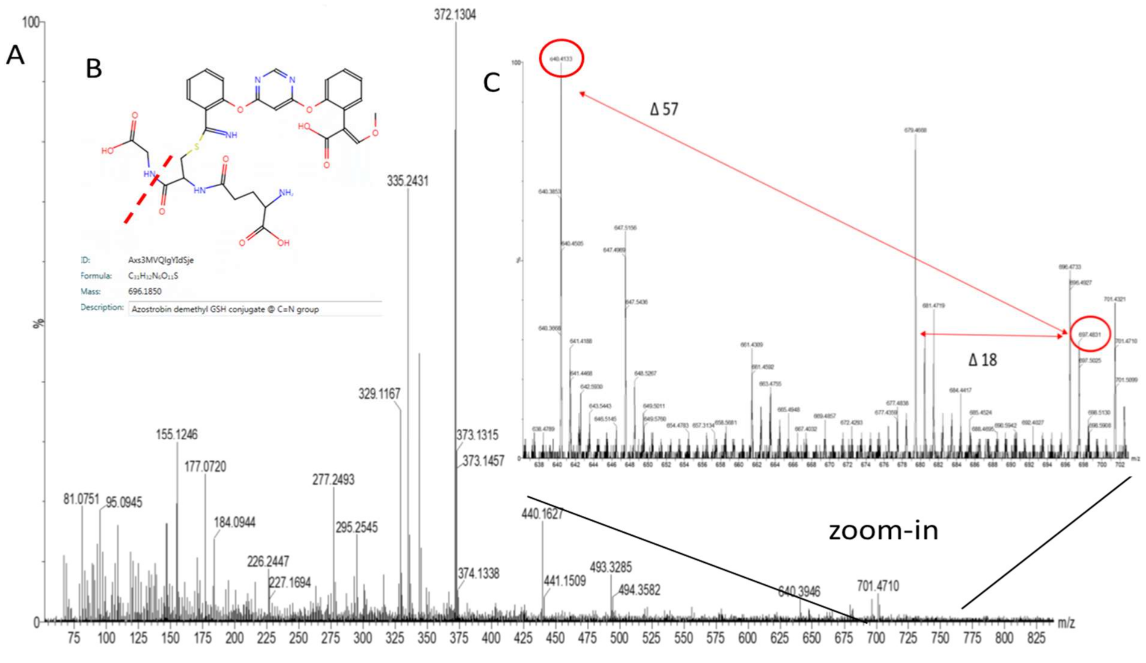

Supplementary Figure 5. Identification of the specie eluted after 30.55 minutes as demethylated azoxystrobin-GSH conjugate of the precursor specie 697.48 m/z. Possible identification of potential demethylated azoxystrobin conjugate at RT 30.55 minutes. The analysis of MS/MS profile of for untreated and treated samples reveals that both 697.48 and 640.41 m/z species are present only in the treated samples. The glycil neutral loss (640.41 m/z is delta 57 from 697.48 m/z) is already occurring at +5eV low energy level (MS1 or MS survey). We have also detected water dehydratation (679.47 is delta 18 from 697.48 m/z) as further confirmation that the compound at RT 30.5 minutes is the demethylated azoxystrobin-GSH conjugate with MW 696.18 (panel B).
